# Remarkable Enhancement of the Hole Mobility in Several Organic Small‐Molecules, Polymers, and Small‐Molecule:Polymer Blend Transistors by Simple Admixing of the Lewis Acid p‐Dopant B(C_6_F_5_)_3_

**DOI:** 10.1002/advs.201700290

**Published:** 2017-10-05

**Authors:** Julianna Panidi, Alexandra F. Paterson, Dongyoon Khim, Zhuping Fei, Yang Han, Leonidas Tsetseris, George Vourlias, Panos A. Patsalas, Martin Heeney, Thomas D. Anthopoulos

**Affiliations:** ^1^ Department of Physics and Centre for Plastic Electronics Imperial College London South Kensington London SW7 2AZ UK; ^2^ Department of Chemistry and Centre for Plastic Electronics Imperial College London South Kensington London SW7 2AZ UK; ^3^ Department of Physics National Technical University of Athens Athens GR‐15780 Greece; ^4^ Department of Physics Laboratory of Applied Physics Aristotle University of Thessaloniki GR‐54124 Thessaloniki Greece; ^5^ Division of Physical Sciences and Engineering King Abdullah University of Science and Technology (KAUST) Thuwal 23955–6900 Saudi Arabia

**Keywords:** doping, Lewis acid dopant, organic semiconductors, organic transistors, printed flexible electronics

## Abstract

Improving the charge carrier mobility of solution‐processable organic semiconductors is critical for the development of advanced organic thin‐film transistors and their application in the emerging sector of printed electronics. Here, a simple method is reported for enhancing the hole mobility in a wide range of organic semiconductors, including small‐molecules, polymers, and small‐molecule:polymer blends, with the latter systems exhibiting the highest mobility. The method is simple and relies on admixing of the molecular Lewis acid B(C_6_F_5_)_3_ in the semiconductor formulation prior to solution deposition. Two prototypical semiconductors where B(C_6_F_5_)_3_ is shown to have a remarkable impact are the blends of 2,8‐difluoro‐5,11‐bis(triethylsilylethynyl)anthradithiophene:poly(triarylamine) (diF‐TESADT:PTAA) and 2,7‐dioctyl[1]‐benzothieno[3,2‐b][1]benzothiophene:poly(indacenodithiophene‐co‐benzothiadiazole) (C8‐BTBT:C16‐IDTBT), for which hole mobilities of 8 and 11 cm^2^ V^−1^ s^−1^, respectively, are obtained. Doping of the 6,13‐bis(triisopropylsilylethynyl)pentacene:PTAA blend with B(C_6_F_5_)_3_ is also shown to increase the maximum hole mobility to 3.7 cm^2^ V^−1^ s^−1^. Analysis of the single and multicomponent materials reveals that B(C_6_F_5_)_3_ plays a dual role, first acting as an efficient p‐dopant, and secondly as a microstructure modifier. Semiconductors that undergo simultaneous p‐doping and dopant‐induced long‐range crystallization are found to consistently outperform transistors based on the pristine materials. Our work underscores Lewis acid doping as a generic strategy towards high performance printed organic microelectronics.

The continuous demand for organic thin‐film transistors (OTFTs) with improved performance has been the driving force behind the tremendous progress witnessed during the past decade in the field of printed electronics.^1^ The most common approach towards this goal has been the development of new semiconductors with enhanced charge transport characteristics^2^, ^3^ accompanied by in‐depth understanding of the material's structure–property relationship and the influence of the all‐important semiconductor/dielectric interface.^4^, ^5^

Amongst the large number of new material systems developed over the past decade, binary semiconducting blends comprised of a small molecule and a conjugated polymer, have proven highly successful primarily due to their remarkable performance and processing versatility.^6^, ^7^, ^8^ It was recently shown that at the heart of this success is the formation of unusually conductive grain boundaries (GBs) that renders this type of blends somewhat immune to the apparent long‐range microstructural variations witnessed via optical microscopy, atomic force microscopy (AFM), transmission electron microscopy (TEM), and scanning electron microscopy (SEM).^9^, ^10^, ^11^ Recent noteworthy achievements include the demonstration of OTFTs based on blends of 2,8‐difluoro‐5,11‐bis(triethylsilylethynyl)anthradithiophene (diF‐TESADT) and the conjugated polymer poly(fluorene‐*co*‐triarylamine) (PF‐TAA) with hole mobility up to 5 cm^2^ V^−1^ s^−1^,^12^ and the development of ternary organic blend OTFTs based on the small molecule 2,7‐dioctyl[1]‐benzothieno[3,2‐b][1]benzothiophene (C_8_‐BTBT), the polymer poly(indacenodithiophene‐co‐benzothiadiazole) (C_16_IDT‐BT), and the molecular p‐dopant C_60_F_48_ for which hole mobility values exceeding to 13 cm^2^ V^−1^ s^−1^ were achieved.^13^ These, together with other recent developments,^14^, ^15^, ^16^, ^17^, ^18^ have catapulted the OTFT performance to a level on par with competing transistor technologies such as metal oxide TFTs.^19^, ^20^

A further noteworthy development in recent years has been the use of molecular doping as a means for improving key operating characteristics of OTFTs including, contact resistance (*R*_C_), charge carrier mobility (μ), bias stress stability, threshold voltage (*V*_TH_), and operating frequency of integrated circuits.^21^, ^22^, ^23^, ^24^, ^25^, ^26^, ^27^, ^28^ There are several methods that one could exploit to dope organic semiconductors with the most studied one being the standard integer charge transfer model.^29^ The latter is based on concepts adopted from inorganic semiconductors and relies on direct charge transfer between a strong electron acceptor/donating molecule and the host semiconductor,^30^ although the details of the mechanism are still debated. The main disadvantage of this model is that it relies on semiconductors with matching energy levels to the dopant, which somewhat limits its applicability. Furthermore, due to significant solubility differences between the two materials, efficient and reliable doping of solution‐processed organic semiconductors has proven challenging.

Recently, an alternative method for p‐doping of certain organic polymers through the addition of a Lewis acid, namely tris(pentafluorophenyl)borane [B(C_6_F_5_)_3_], has been demonstrated.^27^, ^31^, ^32^ A large enhancement in the conductivity of various conjugated polymers was reported, although the doping mechanism remained speculative. Interestingly, the same Lewis acid‐based doping was recently shown to be applicable to polymers with large ionization potential (IP), which otherwise would require dopants with very high electron affinities that are difficult to achieve in practice.^27^ In particular, it was shown that when B(C_6_F_5_)_3_ was admixed with a large IP (>5.7 eV) polymer, the resulting OTFTs exhibited 11‐fold improvement in hole mobility and reduced *V*_TH_, both indicative of p‐doping. The effect was attributed to improved hole injection into the highest occupied molecular orbital (HOMO) of the polymer.

Although the origin of the improved transistor characteristics upon doping depends on various factors,^30^ it is clear that under certain conditions it can lead to profound performance improvement and contribute to the advancement of the OTFT technology. Motivated by these studies, we embarked on investigating the applicability of Lewis acid doping to small‐molecule:polymer blends.^6^ Organic blends offer potential advantages over single materials due to their tunable composition, which could in turn be exploited to enhance the doping efficiency. As the model material system, we have chosen the diF‐TESADT:poly(triarylamine) (PTAA) blend^8^ (**Figure** **1**a). Admixing B(C_6_F_5_)_3_ in the semiconducting blend is found to increase the maximum hole mobility of the transistors from 2.5 to a record value for this mixture of >8 cm^2^ V^−1^ s^−1^. The work was extended to two more blend systems, namely 6,13‐bis(triisopropylsilylethynyl)pentacene (TIPS‐pentacene):PTAA and C_8_‐BTBT:C_16_IDT‐BT, for which similarly pronounced hole mobility enhancement was observed but with an even higher value of ≈11 cm^2^ V^−1^ s^−1^ measured in the latter system. Analysis of the optical, structural, and charge transport properties of the blend systems reveals a dual function for B(C_6_F_5_)_3_; first, acting as an efficient p‐dopant and secondly as a microstructure modifier. Finally, we show that the benefits associated with B(C_6_F_5_)_3_ doping are also applicable to all neat small‐molecules and polymers semiconductors used, further highlighting the generic nature of the doping approach.

**Figure 1:**
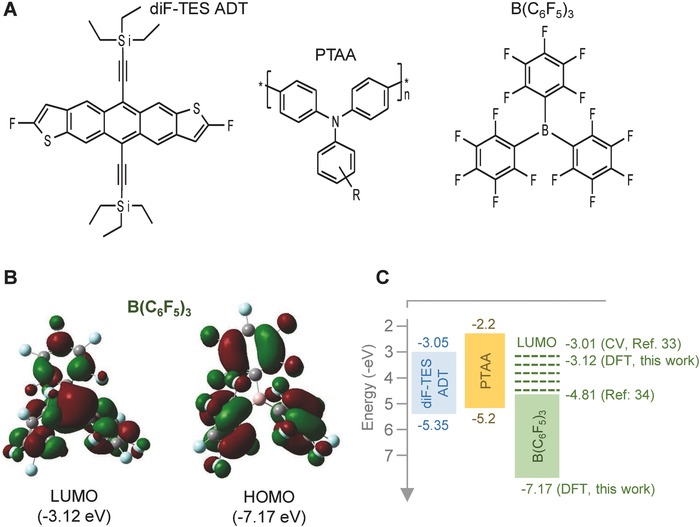
a) Chemical structures of diF‐TESADT, PTAA, and B(C_6_F_5_)_3_. b) Lowest unoccupied molecular orbital (LUMO) and highest occupied molecular orbital (HOMO) energies for B(C_6_F_5_)_3_ calculated via the DFT method. The HOMO is predicted to be localized on phenyl groups while the LUMO to be delocalized over the whole molecule. c) HOMO and LUMO energies of diF‐TESADT, PTAA, and B(C_6_F_5_)_3_. The LUMO energy value for B(C_6_F_5_)_3_ of −3.01 eV was measured via cyclic voltammetry (CV) in ref. 33 while the −4.81 was taken from ref. 34.

Density functional theory (DFT) calculations (Figure 1b) (see the Experimental Section) and cyclic voltammetry data^33^ for the HOMO and lowest unoccupied molecular orbital (LUMO) energies of B(C_6_F_5_)_3_ (3–3.5 eV) suggest a large offset in the energy levels (Figure 1c). Although the latter picture may appear to rule out ground‐state integer electron transfer from diF‐TESADT and/or PTAA to B(C_6_F_5_)_3_, a recent study suggests that such processes may be at play due to the significantly deeper LUMO energy (−4.81 eV) of B(C_6_F_5_)_3_, although it is unclear how this value was derived [see Figure 1c].^34^ Alternatively, Lewis acid/base interactions between B(C_6_F_5_)_3_ and certain chemical species within the blend may take place.^35^ If integer electron transfer took place, it would most likely involve the transfer of an electron from the organic semiconductor(s) to B(C_6_F_5_)_3_ resulting in the formation of radical cation(s) and p‐doping.

To test this hypothesis, we studied the effect of admixing B(C_6_F_5_)_3_ in the diF‐TESADT:PTAA blend on the operating characteristics of bottom‐contact, top‐gate (BC‐TG) OTFTs (**Figure** **2**a, inset). Figure 2a shows the transfer characteristics and corresponding plots of the square root of the channel current (*I*_D_^0.5^) versus gate voltage (*V*_G_), for diF‐TESADT:PTAA:B(C_6_F_5_)_3_ OTFTs with the dopant concentration varying from 0 (pristine) to 3.6 mol%, while Figure S1 (Supporting Information) shows representative output characteristics. Analysis of the data in Figure 2a yields several remarkable findings. First, the hole mobility values, measured both in the linear (μ_h(LIN)_) and saturation (μ_h(SAT)_) regimes, increase considerably with increasing B(C_6_F_5_)_3_ concentration up to 2.4 mol%, for which a maximum value for (μ_h(SAT)_) of up to ≈8 cm^2^ V^−1^ s^−1^ is obtained, followed by a sharp decrease at higher concentrations (Figure 2b). Worth noting at this point is the fact that the latter hole mobility is higher than values reported for single‐crystal diF‐TESADT OTFTs.^5^ Second, the *V*_TH_ reduces with increasing B(C_6_F_5_)_3_ up to 1 mol% beyond which it remains relatively constant (Figure 2c). Thirdly, increasing B(C_6_F_5_)_3_ concentration to >2.4 mol% results to channel off current increase and on/off ratio deterioration (Figure 2d). A further positive effect associated with the presence of B(C_6_F_5_)_3_ is the reduction in the contact resistance (*R*_C_). Figure 2e shows the evolution of *R*_C_ for pristine and B(C_6_F_5_)_3_‐doped (2.4 mol%) OTFTs calculated using the transmission line method,^36^ versus *V*_G_. Although the dependence of *R*_C_ on *V*_G_ for the pristine and doped devices appears similar, B(C_6_F_5_)_3_‐doped OTFTs exhibit significantly reduced *R*_C_. This is an important finding and highlights the important role of *R*_C_ on the mobility enhancement observed (Figure 2b) while indirectly supports the p‐doping effect.^13^, ^37^, ^38^ Further experimental evidence for the latter was obtained by monitoring the work function (φ) and HOMO energy of the blend layer as a function of B(C_6_F_5_)_3_ loading using a combination of Kelvin probe (KP) and air photoemission spectroscopy (APS) (see the Experimental Section). This data is shown in Figure 2f where a clear shift in φ from ≈4.6 eV (pristine blend) to ≈4.75 eV (5 mol% B(C_6_F_5_)_3_) is observed, further supporting the p‐doping effect. On the basis of these results, we conclude that introduction of B(C_6_F_5_)_3_ results to effective *p*‐type doping of the diF‐TESADT:PTAA and to the remarkable reduction in the parasitic contact resistance.

**Figure 2:**
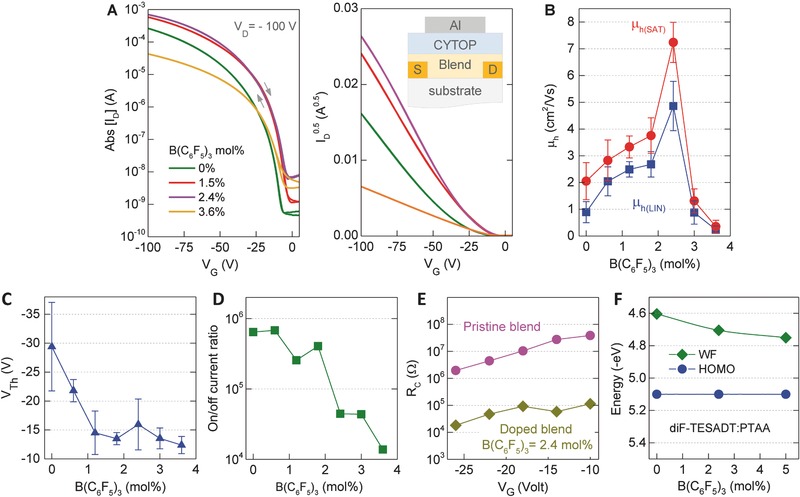
a) Transfer characteristics and corresponding *I*_D_^1/2^ versus *V*_G_ plots measured for top‐gate, bottom‐contact diF‐TESADT:PTAA transistors with different B(C_6_F_5_)_3_ concentrations in the range 0–3.6 mol%. Inset shows the top‐gate, bottom‐contact transistor architecture employed. b) Evolution of hole mobility (*µ*_h_) as a function of B(C_6_F_5_)_3_ concentration. Panels (c) and (d) display the evolution of threshold voltage (*V*_Th_) and current on–off ratio, respectively, as a function of B(C_6_F_5_)_3_ concentration. e) Evolution of contact resistance (*R*_C_) versus *V*_G_ calculated for the pristine and B(C_6_F_5_)_3_(2.4 mol%)‐doped diF‐TESADT:PTAA OTFTs. f) Work function (ϕ) and HOMO energy of the pristine and B(C_6_F_5_)_3_‐doped diF‐TESADT:PTAA blend layers measured by KP and APS, respectively.

To better understand the doping‐induced mobility enhancement mechanism in diF‐TESADT:PTAA:B(C_6_F_5_)_3_ devices, we studied the influence of B(C_6_F_5_)_3_ on the individual semiconductors used, namely PTAA and diF‐TESADT (Figure S2a–d, Supporting Information). PTAA transistors exhibit progressively enhanced p‐doping with increasing B(C_6_F_5_)_3_ concentration manifested as increased channel off‐current (Figure S2a, Supporting Information), while the on‐current reduces slightly. On the other hand, both μ_h(SAT)_ and μ_h(LIN)_ remain relatively independent to B(C_6_F_5_)_3_ concentration up to 2 mol%, with the former reducing at higher concentrations. The values of μ_h(SAT)_ at >2 mol% doping could not be determined due to the inability of the device to enter that pinch‐off regime—a direct result of the high background hole concentration. The transformation of the clear PTAA solution to brightly colored one upon B(C_6_F_5_)_3_ addition, indicates strong interaction between PTAA and B(C_6_F_5_)_3_, which supports the p‐doping effect seen in Figure S2A in the Supporting Information. However, considering the absence of strongly basic groups on PTAA, one can argue that the formation of Lewis acid–‐base adducts (supramolecular charge transfer complex) may not be the dominant process responsible for the p‐doping effect and that B(C_6_F_5_)_3_ is acting as an oxidant. Similar oxidation processes involving B(C_6_F_5_)_3_ have been reported for other arylamine based materials, and as such remains a plausible mechanism that cannot be currently excluded.^35^ The impact of B(C_6_F_5_)_3_ on hole transport in pristine diF‐TESADT transistors (Figure S2c, Supporting Information) is somewhat similar to PTAA devices but with the mobility exhibiting a significant initial increase, which is attributed primarily to the enhanced channel transconductance, followed by a sharp drop for dopant concentrations >2 mol% due to the noticeable reduction in the channel current. A gradual increase in the channel off‐current is also observed with increasing dopant concentration indicating efficient p‐doping. Similar to PTAA, the coloration of the diF‐TESADT solution changes from light orange to deep red upon addition of B(C_6_F_5_)_3_ (Figure S2d, Supporting Information) revealing the existence of strong interactions between the two materials. Unlike PTAA, however, preliminary DFT calculations suggest that B(C_6_F_5_)_3_ could indeed form adduct complexes via B—C bonds to diF‐TESADT (see the Supporting Information). These results support the existence of strong interactions between B(C_6_F_5_)_3_ with diF‐TESADT and PTAA and most likely underpin the p‐doping effect. One may also conclude that the 4‐fold mobility enhancement observed in the p‐doped diF‐TESADT:PTAA devices can only be attributed to interactions between B(C_6_F_5_)_3_ and diF‐TESADT since the mobility of PTAA is far too low to have a noticeable impact on the overall charge transport across the channel even when p‐doped.

The influence of B(C_6_F_5_)_3_ on the blend layer microstructure was also investigated. **Figure** **3**a,b shows the polarized optical microscopy images for the pristine and B(C_6_F_5_)_3_‐doped diF‐TESADT:PTAA layers processed via spin coating at room temperature. Although the images appear similar, closer analysis of their surfaces via atomic force microscopy (AFM) reveals significant differences. Figure 3c,d displays the AFM images of the surface topography of the pristine and B(C_6_F_5_)_3_‐doped (2.4 mol%) films, respectively, while Figure 3e shows the height histograms extracted from the AFM data. The latter figure is of particular significance as it reveals the existence of two very distinct surface topographies. Pristine diF‐TESADT:PTAA layers exhibit two broad peaks, one centered at ≈5.8 nm (peak 1) and another at ≈30 nm (peak 2). The former is attributed to the flat regions present on the surface of the film highlighted by box 1 in Figure 3c, whereas the latter to regions with large height variation (box 2). Contrariwise, the B(C_6_F_5_)_3_‐doped diF‐TESADT:PTAA:B(C_6_F_5_)_3_(2.4 mol%) layer exhibits well‐defined multimodal height distributions indicative of the presence of molecularly flat crystalline plateaus. The height of these terraces are centered at ≈8 nm (peak 1), ≈16 nm (peak 2), and ≈31 nm (peak 3). These features are clearly visible in Figure 3d where the numbered boxes highlight areas representative of each peak/plateau seen in Figure 3e [B(C_6_F_5_)_3_‐doped blend]. The cartoons in Figure 3f represent simplified illustrations of the two surface topographies that would give rise to height histograms qualitatively similar to those shown in Figure 3e. The AFM data reveal the remarkable impact of B(C_6_F_5_)_3_ on the surface morphology of the diF‐TESADT:PTAA layer. The most dramatic feature is the formation of molecular terraces with the plateaus extending to >10 µm, i.e., distances comparable to the channel length of the transistors employed.

**Figure 3:**
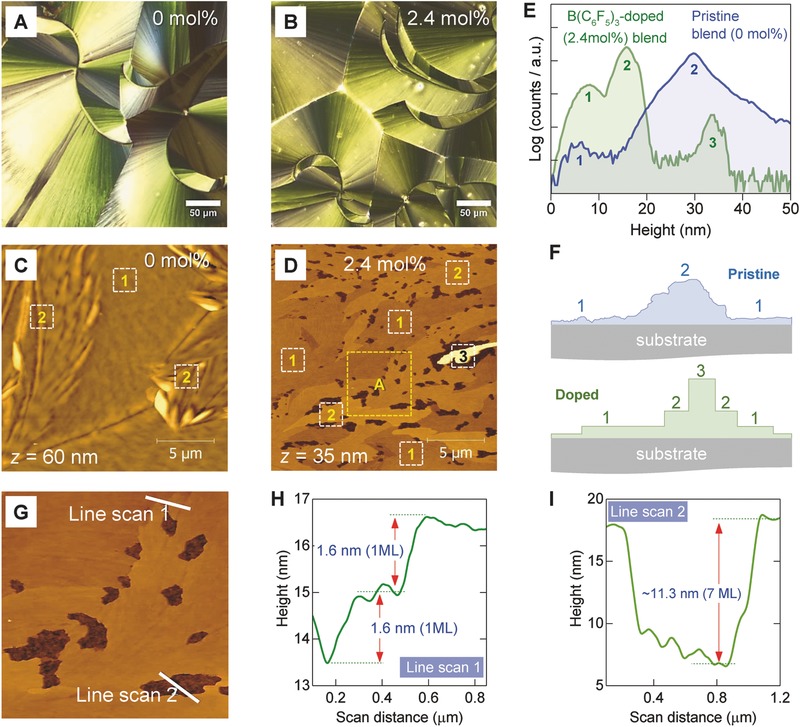
Polarized optical microscopy images of a) a pristine (0 mol%) and b) a B(C_6_F_5_)_3_‐doped (2.4 mol%) diF‐TESADT:PTAA blend layer. Topographical AFM images of c) a pristine (0 mol%) and d) a B(C_6_F_5_)_3_‐doped (2.4 mol%) diF‐TESADT:PTAA layer, and e) their corresponding height histograms. f) Cartoons representing schematics of the layer cross‐section of the pristine and B(C_6_F_5_)_3_‐doped diF‐TESADT:PTAA layers. g) Higher magnification AFM image of region A (box) highlighted in panel (d). Line scans h) 1 and i) 2 obtained from the AFM image in panel (g).

The crystalline molecularly flat domains seen in the B(C_6_F_5_)_3_‐doped blend in Figure 3d resemble molecular terraces reported previously for polycrystalline layers^39^ and single crystals of diF‐TESADT.^5^ Here, however, the terraces are multiples of the *d*_001_ spacing (16.3 Å) of the triclinic crystal of diF‐TESADT ($P\bar{1}$ space group).^5^, ^40^ Specifically, peak 1 in the B(C_6_F_5_)_3_‐doped layer in Figure 3e centered at ≈8 nm corresponds to an average terrace height profile of five monomolecular layers assuming a ML height of ≈16 Å—a value close to the *d*_001_ spacing (16.3 Å) of the triclinic diF‐TESADT crystal.^5^ Similarly, peaks 2 and 3 represent terraces comprised of 10 and 20 monomolecular layers, respectively. In fact, analysis of the highlighted region (box A) in Figure 3d, shown as Figure 3g, reveals the coexistence of single monolayers (1 ML) ≈1.6 nm in height (Figure 3h, line scan 1) and multiple monomolecular terraces (Figure 3i, line scan 2), in agreement with Figure 3e. We thus conclude that diF‐TESADT:PTAA:B(C_6_F_5_)_3_(2.4 mol%) layers exhibit long‐range molecular terracing with the *c*‐axis of the crystal oriented orthogonal to the substrate and the *a*–*b* plane parallel to it. Since these features are only present in the B(C_6_F_5_)_3_‐doped blend layers, we propose that addition of B(C_6_F_5_)_3_ in diF‐TESADT:PTAA mediates the small‐molecule crystallization with profound impact on the layer microstructure. The dramatic improvement in the crystallinity of the upper phase‐separated diF‐TESADT layer in the diF‐TESADT:PTAA:B(C_6_F_5_)_3_(2.4%) blend is arguably expected to play an important role in the mobility enhancement seen in Figure 2b.

To gain more insights into the impact of B(C_6_F_5_)_3_ on the diF‐TESADT:PTAA blend microstructure, we performed X‐ray diffraction (XRD) analysis in Bragg–Brentano geometry. **Figure** **4**a shows the obtained diffractograms for the four samples studied, namely, diF‐TESADT, diF‐TESADT:B(C_6_F_5_)_3_, diF‐TESADT:PTAA, and diF‐TESADT:PTAA:B(C_6_F_5_)_3_(2.4 mol%), while Figure S3 (Supporting Information) displays the raw data prior to background subtraction. With the exception of PTAA, which is entirely amorphous (data not shown), all four samples exhibit crystalline features. The strong diffraction peak at 2.72–2.74° corresponds to the *d*_001_ spacing (1.612–1.625 nm) of the triclinic ($P\bar{1}$ space group) crystal of diF‐TESADT^41^ and confirms the AFM observations of molecular terracing. Secondary peaks at wider angles are also detected and correspond to the (00h) family of planes suggesting a (001) lamellar structure parallel to the substrate surface. In order to quantify the XRD data, we fitted the three prominent (00h) diffraction peaks for each sample by Voigt curves each consisting of a Lorentzian and a Gaussian contribution. The Lorentzian contribution provides information to variations in the vertical grain size (*G*_z_) while the Gaussian contribution relates to microstrain (ε_s_), which in turn relates to structural defects and the microscopic strain field around them.^42^ For the purpose of this study, we chose to analyze the (003) diffraction peaks as they offer the best compromise between the intrinsic angular resolution limits of the XRD measurements and the signal‐to‐noise ratio. A Lorentzian broadening (*W*_L_) was introduced into Scherrer's formula to determine *G*_z_ along the *z*‐direction, while a Gaussian broadening (*W*_G_) was used to estimate microstrain given by ε_s_ = (*W*_G_ × cosθ/4 × sinθ). Note that the instrumental broadening (<0.1^o^) was not taken into account in our calculations. Given that the observed XRD peaks are exceptionally sharp, neglecting the instrumental broadening might result in underestimation of the vertical grain size and overestimation of the microstrain. Nevertheless, the qualitative relative values of these quantities for the various samples of this study are still valid.

**Figure 4:**
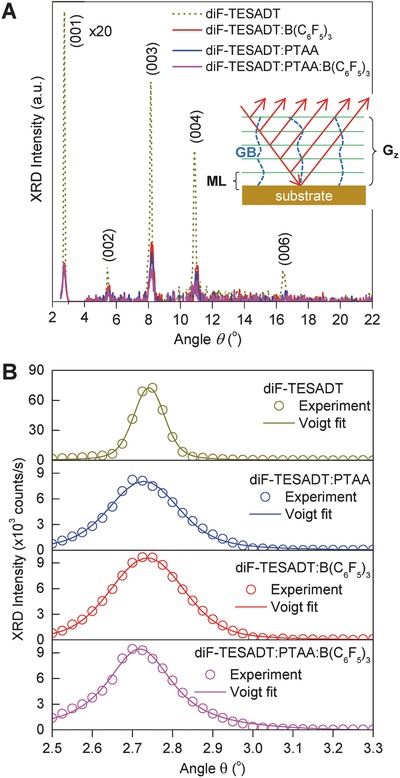
a) X‐ray diffractograms for the four samples studied namely, diF‐TESADT, diF‐TESADT:B(C_6_F_5_)_3_, diF‐TESADT:PTAA, and diF‐TESADT:PTAA:B(C_6_F_5_)_3_(2.4 mol%). b) X‐ray diffractograms around the (001) peak (symbols) and the corresponding Voigt fits.

The inset in Figure 4a illustrates the origin of microstrain in the case of lamellar layer morphology due to the presence of GBs. As the diffracted X‐rays are propagating through the layer they interact with the strained regions surrounding the GBs. When the density of GBs in the sampling volume increases, the contribution of the strained regions to the XRD signal is expected to increase too. Thus, ε_s_ and *G*_z_ can be used as indicators of the lateral and out‐of‐plane layer crystallinity, respectively. Figure 4b shows details of the X‐ray diffractograms around the (001) peak and the corresponding Voigt fits. The quantitative results are summarized in Tables S1–S4 and Figures S4–S6 in the Supporting Information. Data analysis reveals that diF‐TESADT:PTAA layers exhibit inferior crystallinity, both out of plane (small *G*_z_) and laterally (large ε_s_), as compared to neat diF‐TESADT layers. Doped diF‐TESADT:PTAA:B(C_6_F_5_)_3_ (2.4 mol%) layers, on the other hand, yield substantially reduced *G*_z_ and ε_s_ values suggesting the existence of thinner layers with enhanced lateral long‐range crystallinity. This picture is in good agreement with the molecular terracing seen in the AFM images in Figure 3g–i. It also suggests that diF‐TESADT:PTAA:B(C_6_F_5_)_3_ (2.4 mol%) layers contain fewer GBs, which are known to be associated with hole trap states,^43^ and hence contribute to the improved OTFT performance seen in Figure 2a,b for B(C_6_F_5_)_3_ concentration of 2.4 mol%.

To investigate whether the Lewis acid doping approach is applicable to other blends, we extended our study to two more systems namely, C_8_‐BTBT:C_16_IDT‐BT (**Figure** **5**a)^13^ and TIPS‐pentacene:PTAA (Figure 5b).^8^ Figure 5c shows the AFM images of the surface topography of a pristine and a B(C_6_F_5_)_3_ (0.05 mol%)‐doped C_8_‐BTBT:C_16_IDT‐BT layers. Analysis of the height histograms shown in Figure 5d reveals significant differences between the two surfaces. The pristine layer exhibits broader height distribution, as compared to the B(C_6_F_5_)_3_(0.05 mol%)‐doped system, with the latter being characterized by a smoother surface and several distinct peaks indicative of molecular terracing. Admixing of B(C_6_F_5_)_3_(2.4 mol%) in the TIPS‐pentacene:PTAA blend has a similar “smoothing” effect on the layer's surface topography and can clearly be seen in the AFM images and corresponding height histograms in Figure 5e,f, respectively. However, unlike C_8_‐BTBT:C_16_IDT‐BT:B(C_6_F_5_)_3_ (0.05 mol%), we see no evidence of molecular terracing with the surface topographies, for both pristine and B(C_6_F_5_)_3_‐doped TIPS‐pentacene:PTAA blend layers, exhibiting broad height distributions centered at ≈26 and ≈30 nm, respectively.

**Figure 5:**
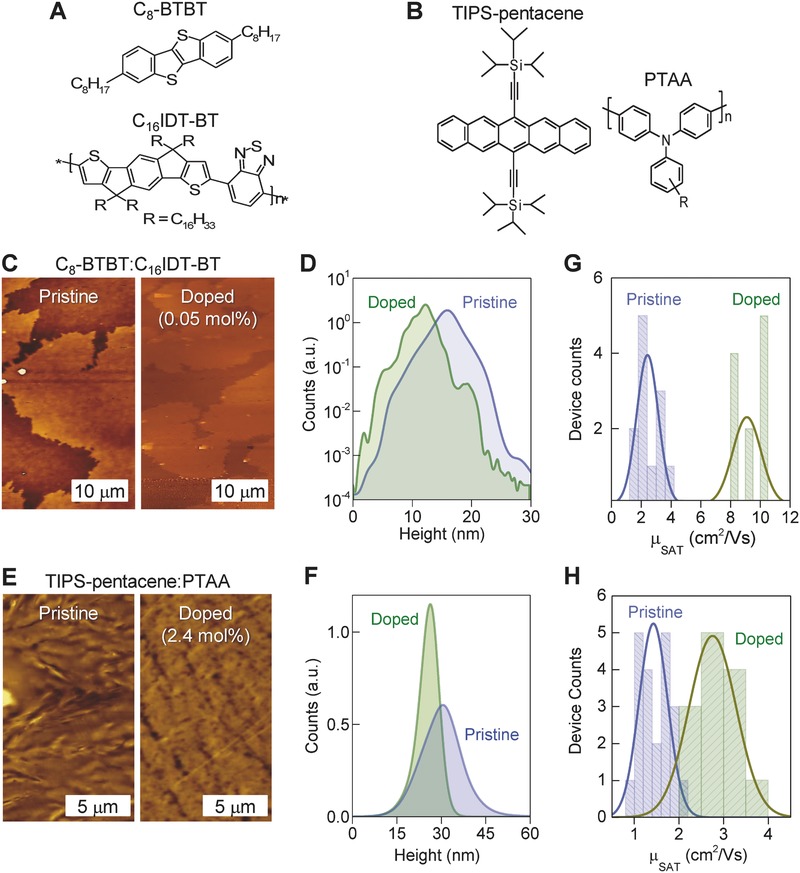
a,b) Chemical structures of the materials used in the two additional semiconducting blends. c) Topography AFM images of the pristine (0 mol%) and B(C_6_F_5_)_3_‐doped (0.05 mol%) layers composed of C_8_‐BTBT:C_16_IDT‐BT and d) the corresponding height histograms. e) Topography AFM images of the pristine (0 mol%) and B(C_6_F_5_)_3_‐doped (2.4 mol%) TIPS‐pentacene:PTAA layers and f) the corresponding height histograms. Panels (g) and (h) show the extracted field‐effect hole mobility measured in saturation (µ_SAT_), for the pristine and B(C_6_F_5_)_3_‐doped C_8_‐BTBT:C_16_IDT‐BT (g) and TIPS‐pentacene:PTAA (h) transistors.

The impact of B(C_6_F_5_)_3_ in the hole transport properties of C_8_‐BTBT:C_16_IDT‐BT and TIPS‐pentacene:PTAA blends was also investigated. C_8_‐BTBT:C_16_IDT‐BT transistors exhibit excellent operating characteristics (Figure S7a, Supporting Information) with the pristine blend yielding hole mobility values in the range of 1–4 cm^2^ V^−1^ s^−1^ (Figure 5d). The addition of B(C_6_F_5_)_3_ leads to a significant improvement in hole transport (Figure S7b, Supporting Information) with the mobility now reaching values up to 11 cm^2^ V^−1^ s^−1^ (Figure 5g). A similar but less pronounced effect is observed for TIPS‐pentacene:PTAA transistors for which a maximum hole mobility value of ≈3.7 cm^2^ V^−1^ s^−1^ is obtained upon doping with 2.4 mol% B(C_6_F_5_)_3_ (Figure 5h; Figure S8, Supporting Information). Hardly any current hysteresis in the transfer characteristics is observed for either blend indicating a good quality semiconductor/dielectric interface. Direct experimental evidence of efficient p‐doping was obtained via KP measurements performed on both pristine and B(C_6_F_5_)_3_‐doped blends (Figure S9, Supporting Information). The Fermi energy is found to shift towards the HOMO energy by ≈0.56 eV, for C_8_‐BTBT:C_16_IDT‐BT (Figure S9a, Supporting Information), and by ≈0.41 eV for TIPS‐pentacene:PTAA (Figure S9b, Supporting Information), upon 5 mol% doping.

Further insights into the inner working of the doping process in these blends were obtained by studying the influence of B(C_6_F_5_)_3_ on the hole transporting properties of the individual semiconductors, namely C_8_‐BTBT (Figure S10a,b, Supporting Information), C_16_IDT‐BT (Figure S10c,d, Supporting Information), and TIPS‐pentacene (Figure S10e,f, Supporting Information). The hole mobility in all devices was found to exhibit a characteristic maximum at a specific B(C_6_F_5_)_3_ concentration, the value of which slightly differs for each material, followed by a roll‐off at higher concentrations. The latter is attributed to the deterioration of the channel transconductance, and in the case of TIPS‐pentacene, to the overall reduction of the channel current (Figure S10e, Supporting Information). We note, however, that calculation of μ_h(SAT)_ at high doping concentration for certain materials becomes problematic as the resulting OTFTs cannot be operated in saturation. For this reason, some of the μ_h(SAT)_ values are not included in these plots. Finally, the off‐current in all transistors was found to increase with increasing B(C_6_F_5_) concentration indicating efficient p‐doping for all materials studied.

The experimental data presented so far indicate that B(C_6_F_5_)_3_ interacts strongly with all semiconductors considered directly affecting the nucleation and growth of all studied systems. As noted earlier, a possible process that could facilitate these interactions is the formation of adduct complexes. Given the diverse chemical properties of the materials studied, however, the identification of such complexes is a non‐trivial task. For this matter, total energy DFT calculations provide an indispensable tool to examine several different possibilities for adduct formation. Preliminary results on the stability of various complexes (either in physisorbed or chemisorbed configurations) confirm that B(C_6_F_5_)_3_ could indeed form adduct complexes via B—C bonds to some of the molecules studied and particularly diF‐TESADT and TIPS‐pentacene. Likewise, adduct formation through B—N bonds is favored between B(C_6_F_5_)_3_ molecules and C_16_IDT‐BT polymer chains. The energies of these chemisorbed structures (Figure S11, Supporting Information) are calculated to be less than 0.07 eV but higher than those of corresponding physisorbed configurations. These findings provide further evidence for the potential dual role of B(C_6_F_5_)_3_, namely that it not only affects the long‐range microstructure of the semiconducting layer but also binds at the molecular level to certain species of interest. On the other hand, it should be noted that we have not been able to identify any stable adduct structures involving B(C_6_F_5_)_3_ and C_8_‐BTBT molecules or PTAA polymer chains. Therefore, in these latter two cases, either there are some unaccounted complexes and/or other species present within the material/blend (impurities, chemicals defects, etc.), contribute to chemical interactions and to the apparent p‐doping. However, detailed analysis of such processes is beyond the scope of this work and will be the subject of future studies.

In conclusion, we studied the influence of admixing the Lewis acid B(C_6_F_5_)_3_ in several p‐type organic semiconductors including, small‐molecules, polymers, and small‐molecule:polymer blend systems. Using a range of characterization techniques and DFT calculations, we showed that addition of B(C_6_F_5_)_3_ can result to effective *p*‐doping and, under certain conditions, to remarkable improvements of the hole mobility in several of these semiconducting materials and their small‐molecule:polymer blends. A further important discovery was the positive impact of B(C_6_F_5_)_3_ on the microstructure of all small‐molecule:polymer blend layers investigated with certain systems exhibiting long‐range molecular terracing—i.e., features similar to those observed in single crystals of the small‐molecule components employed. Our findings suggest that semiconducting blends that undergo simultaneous p‐doping and dopant‐induced long‐range crystallization consistently outperform transistors based on pristine blends. Two prototypical blend semiconductors where the B(C_6_F_5_)_3_ doping was shown to have a remarkable impact are those of diF‐TESADT:PTAA and C_8_‐BTBT:C_16_IDT‐BT for which hole mobilities up to ≈8 and ≈11 cm^2^ V^−1^ s^−1^, respectively, were obtained. Doping of the TIPS‐pentacene:PTAA blend with B(C_6_F_5_)_3_ also resulted to a significantly enhanced mobility but with a maximum value of 3.7 cm^2^ V^−1^ s^−1^. Analysis of the data indicates that reduction of the contact resistance due to p‐doping by B(C_6_F_5_)_3_ and the accompanied improvement of the surface's long‐range crystallinity are the two primary features responsible for the doping‐induced hole mobility enhancement. The findings underscore Lewis acid doping as an alternative strategy for the development of high‐charge carrier mobility organic semiconductors, transistors, and integrated circuits manufactured using solution‐phase deposition processes.

## Experimental Section

*DFT Calculations*: The energy distributions and energy levels of B(C_6_F_5_)_3_ were evaluated by quantum mechanical calculations at the B3LYP/6‐31 G* level of theory performed using Gaussian 09. Total energy calculations were performed with the DFT code Quantum Espresso,^44^ including van der Waals interactions within the DFT‐D2 approach^45^ and exchange–correlation effects with the Perdew–Wang functional.^46^ The energy cutoff for the plane wave basis was set at 75 Rydbergs, and the interaction between valence electrons and ions was described with projector‐augmented waves.^47^

*Solution Preparation*: The small‐molecule polymer blend systems diFTESADT:PTAA, TIPS‐pentacene:PTAA were prepared at 1:1 wt%, while the C8‐BTBT:C16IDT‐BT at 1:3 wt% ratio by following previously reported procedures using tetralin and/or chlorobenzene as the solvents.^8^, ^9^, ^13^ The dopant species were added from a 10 × 10^−3^ m B(C_6_F_5_)_3_ solution in 1,2‐dichlorobenzene for diF‐TESADT:PTAA and TIPS‐pentacene:PTAA blends, while 5 × 10^−3^ m of B(C_6_F_5_)_3_ in 1,2‐dichlorobenzene was used for the C_8_‐BTBT:C_16_IDT‐BT blend. The required amount of solution was then added to the semiconducting blend formulation. The dopant concentration was calculated with respect to the molarity of the small‐molecule component (mol%). Small‐molecule transistors were prepared from 5 mg mL^−1^ of diF‐TESADT and TIPS‐pentacene and 2 mg mL^−1^ of C_8_‐BTBT in chlorobenzene. In this case, 5 × 10^−3^ m B(C_6_F_5_)_3_ in chlorobenzene was used for TIPS‐pentacene and C_8_‐BTBT, and 10 × 10^−3^ m for diF‐TESADT. C_16_IDT‐BT solutions were prepared from 2 mg mL^−1^ in chlorobenzene, and PTAA from 10 mg mL^−1^ in toluene. For both polymers, the dopant was a 5 × 10^−3^ m solution in chlorobenzene calculating the mol% with respect to the repeat unit of the polymers.

*Transistor Fabrication and Characterization*: Bottom‐contact, top‐gate (BC–TG) configuration TFTs were used to identify the doping effect in the blends/small‐molecule/polymer organic semiconductors. Glass substrates were cleaned by sonication in a detergent solution of Decon 90, followed by rinse with acetone and isopropanol. Gold source–drain (S–D) electrodes (40 nm) were deposited via thermal evaporation in high vacuum (10^−6^ mbar) through shadow masks. The S–D electrodes containing substrates were then immersed in a pentafluorothiophenol (PFBT) self‐assembled monolayer (SAM) solution for 10 min to modify the work function of gold. The semiconductor blend films were spin cast in two steps: 500 rpm for 10 s followed by 2000 rpm for 20 s. Annealing the diF‐TESADT:PTAA and TIPS‐pentacene:PTAA was performed at 100 °C for 15 min, while the C_8_‐BTBT:C_16_IDT‐BT was annealed at 120 °C for 2 min. For the small molecule and polymer TFTs, semiconductors were spin coated at 2000 rpm for 30 s. The films were annealed for 5 min at 100 °C. 900 nm CYTOP was used as a dielectric layer and the gate electrodes were deposited by thermal evaporation of 40 nm aluminum through shadow masks. Electrical characterization of all transistors was conducted in a dry nitrogen glovebox using an Agilent B2902A semiconductor parameter analyzer.

*X‐Ray Diffraction Measurements*: Semiconductor materials were deposited on doped Si^++^ substrates via spin coating. X‐Ray diffraction pattern was obtained in θ–θ geometry using a Rigaku Ultima diffractometer equipped with a Cu anode and an X‐ray monochromator (λ = 1.54 Å).

*Kelvin Probe and Air Photoemission Spectroscopy*: Organic semiconductor blends were deposited on Indium Tin Oxide (ITO) substrates in order to measure their work function and HOMO energy level by a KP Technology, SKP5050/APS02 set up. Silver was used as reference sample for both KP and APS. For the photoemission experiment, the incident light was scanning from 6 to 4eV recording the photocurrent response.

*Atomic Force Microscopy*: AFM was carried out in a taping mode using an Agilent 5500 to map topography, phase, and amplitude of the semiconductor blend layers. Statistical data were then extracted using the Gwyddion software.

*Polarized Optical Microscopy*: A Nikon Eclipse E600 POL was used to image the films between crossed polarizers.

## Conflict of Interest

The authors declare no conflict of interest.

## Supporting information

Supplementary
