# Supplementary material for: Remarkable Enhancement of the Hole Mobility in Several Organic Small‐Molecules, Polymers, and Small‐Molecule:Polymer Blend Transistors by Simple Admixing of the Lewis Acid p‐Dopant B(C6F5)3
Source: Adv Sci (Weinh). 2017 Oct 5;5(1):1700290. doi: 10.1002/advs.201700290 (PMC5770661; doi:10.1002/advs.201700290)
Supplement: Supplementary file 1 — Supplementary [file ADVS-5-na-s001.pdf]

Copyright WILEY-VCH Verlag GmbH & Co. KGaA, 69469 Weinheim, Germany, 2017.

ADVANCED SCIENCE
Open Access

Supporting Information

for *Adv. Sci.,* DOI: 10.1002/advs.201700290

Remarkable Enhancement of the Hole Mobility in Several Organic Small-Molecules, Polymers, and Small-Molecule:Polymer Blend Transistors by Simple Admixing of the Lewis Acid p-Dopant $B(C_6F_5)_3$

*Julianna Panidi, Alexandra F. Paterson, Dongyoon Khim, Zhuping Fei, Yang Han, Leonidas Tsetseris, George Vourlias, Panos A. Patsalas, Martin Heeney, and Thomas D. Anthopoulos**

Copyright WILEY-VCH Verlag GmbH & Co. KGaA, 69469 Weinheim, Germany, 2016.

Supporting Information

Remarkable enhancement of the hole mobility in several organic small-molecules, polymers and small-molecule:polymer blend transistors by simple admixing of the Lewis acid p-dopant $B(C_6F_5)_3$

*Julianna Panidi, Alexandra F. Paterson, Dongyoon Khim, Zhuping Fei, Yang Han, Leonidas Tsetseris, George Vourlias, Panos A. Patsalas, Martin Heeney, and Thomas D. Anthopoulos**

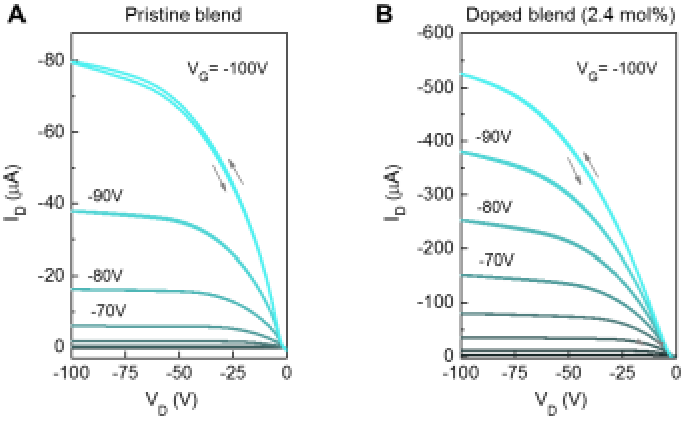

Figure S1. Output characteristics obtained for OTFTs based on, (a) pristine diF-TESADT:PTAA, and (b) diF-TESADT:PTAA:$B(C_6F_5)_3$(2.4 mol%) (doped) semiconducting layers. The channel length and width of both OTFTs were W = 1 mm and L = 40 µm.

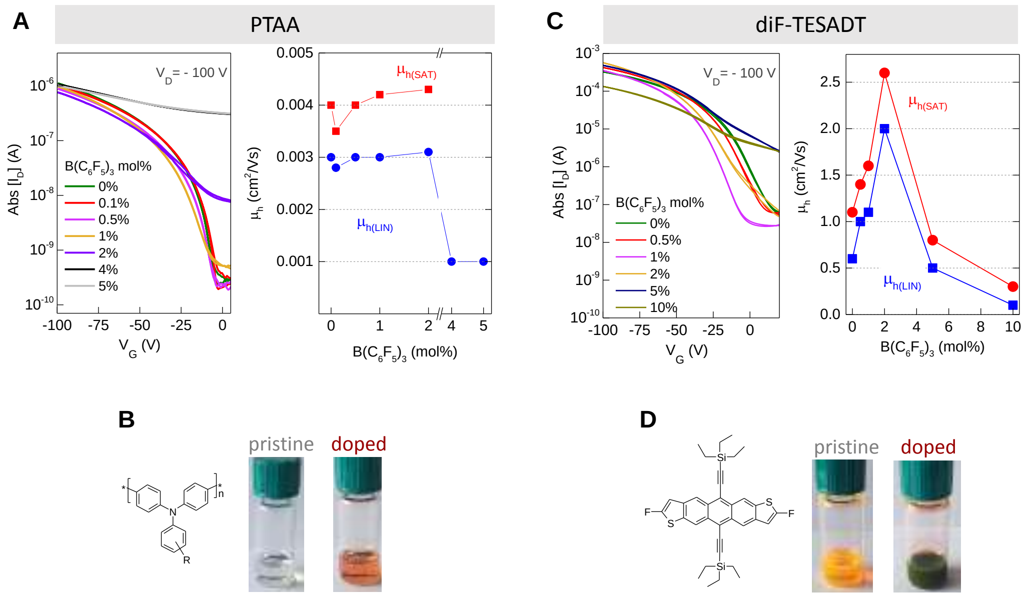

Figure S2. (a) Transfer characteristics and hole mobility (μ_h) of OTFTs based on pristine and $B(C_6F_5)_3$-doped PTAA. (b) Photograph of the PTAA solution before and after $B(C_6F_5)_3$ addition (5% mol). (c) Transfer characteristics and hole mobility (μ_h) of OTFTs based on pristine and $B(C_6F_5)_3$-doped diF-TESADT. (d) Photograph of the diF-TESADT solution before and after $B(C_6F_5)_3$ addition (5% mol).

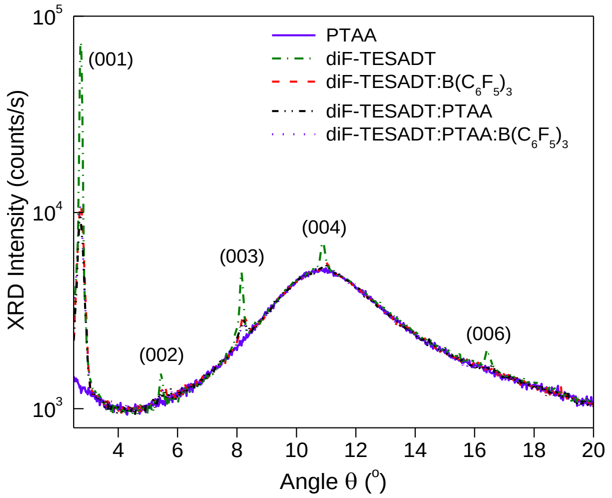

Figure S3. Raw XRD data of five reference samples (PTAA, diF-TESADT, diF-TESADT:B(C_6F_5)$_3$, diF-TESADT:PTAA, and diF-TESADT:PTAA:B(C_6F_5)$_3$. For all samples, there is a wide peak cantered at 11°, which is due to the glass substrate. The contribution of the glass substrate was subtracted from the XRD scans in order to have a more precise view of the X-ray diffractograms of the measured samples. The quantitative results of the XRD analysis of the three stronger peaks are presented in **Tables S1-S4**. Note that the signal-to-noise ratio of the (004) peak of the diF-TESADT:B(C_6F_5)$_3$ sample was not adequate to fit.

Table S1: The Bragg angles of the three stronger peaks of the films.

Sample	(001) Bragg angle (^{o})	(003) Bragg angle (^{o})	(004) Bragg angle (^{o})
diF-TESADT	2.74126	8.14911	10.89099
diF-TESADT:B(C_6F_5)$_3$	2.73683	8.22051	11.01698
diF-TESADT:PTAA	2.72819	8.19169	10.99867
diF-TESADT:PTAA:B(C_6F_5)$_3$	2.7183	8.16899	N/A

Table S2: The *d*-spacing of the three stronger peaks of the films; in parentheses are the equivalent values of the d_{001}.

Sample	(001) *d*-spacing (nm)	(003) *d*-spacing (nm)	(004) *d*-spacing (nm)
diF-TESADT	1.61189	0.54321 (1.62963)	0.40753 (1.63012)
diF-TESADT:B(C_6F_5)$_3$(2.4 mol%)	1.6145	0.53852 (1.61556)	0.40293 (1.61172)
diF-TESADT:PTAA	1.61961	0.54041 (1.62123)	0.40359 (1.61436)
diF-TESADT:PTAA:B(C_6F_5)$_3$(2.4 mol%)	1.6255	0.5419 (1.62570)	N/A

Table S3: The vertical grain size G_z determined from the three stronger peaks of the films. Note that with increasing order of diffraction the accuracy of the calculation is substantially improved. G_z is an indicator of the vertical crystallinity.

Sample	(001) G_z (nm)	(003) G_z (nm)	(004) G_z (nm)

diF-TESADT	269.5928	50.04775	57.50282
diF-TESADT:B(C_6F_5)$_3$ (2.4 mol%)	130.9763	36.40266	54.08023
diF-TESADT:PTAA	84.66673	27.16047	12.48876
diF-TESADT:PTAA:B(C_6F_5)$_3$ (2.4 mol%)	44.16745	13.96916	N/A

Table S4: The microstrain ε_s determined from the three stronger peaks of the films. Note that with increasing order of diffraction the accuracy of the calculation is substantially improved. ε_s is an indicator of the lateral crystallinity.

**Sample**	**(001) ε_s**	**(003) ε_s**	**(004) ε_s**
diF-TESADT	0.00642	0.00481	0.02201
diF-TESADT:B(C_6F_5)$_3$ (2.4 mol%)	0.01686	0.03805	0.03792
diF-TESADT:PTAA	0.01399	0.05925	6.46×10^{-8}
diF-TESADT:PTAA:B(C_6F_5)$_3$ (2.4 mol%)	0.00505	1.94×10^{-16}	N/A

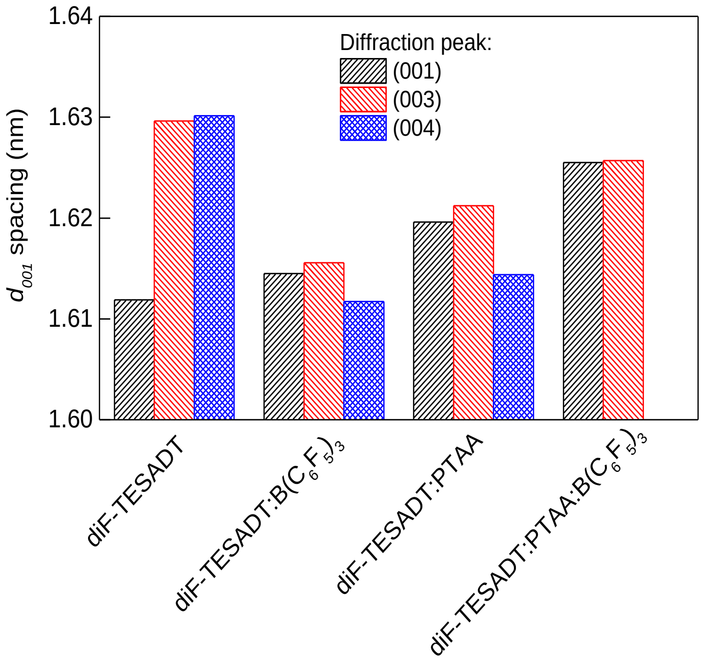

Figure S4. The d_{001} spacing for the four crystalline samples as determined by XRD peak profile analysis of the (001), (003) and (004) XRD peaks.

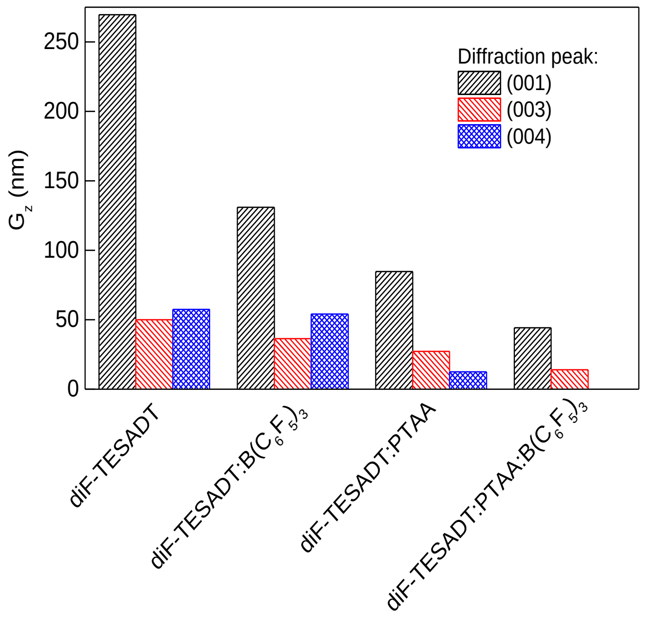

Figure S5. The vertical grain size for the four crystalline samples as determined by XRD peak profile analysis.

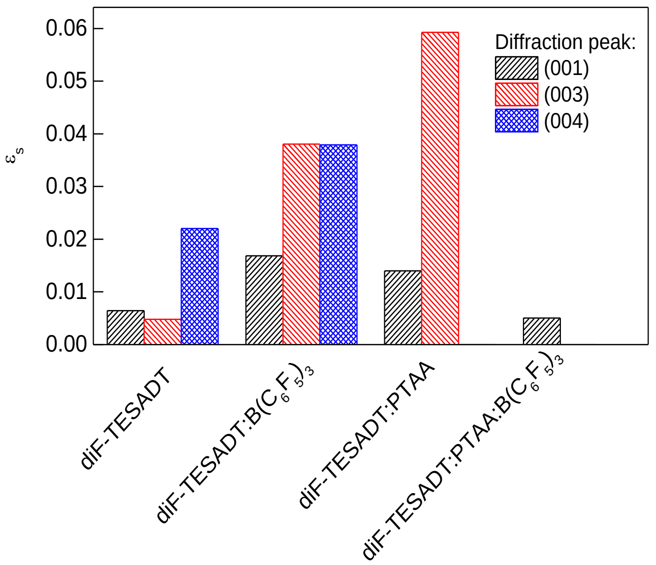

Figure S6. Microstrain (ε_S) for the four polycrystalline samples as determined by XRD peak profile analysis.

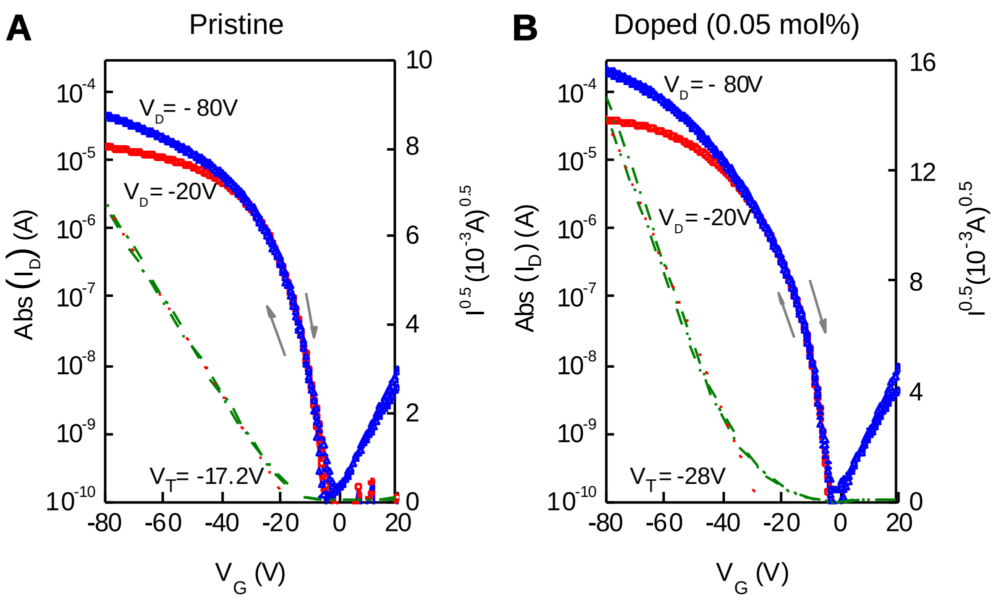

Figure S7. Transfer characteristics measured for OTFTs based on: (a) $C_8BTBT:C_{16}IDT\text{-}BT$ (pristine), and (b) $C_8BTBT:C_{16}IDT\text{-}BT:B(C_6F_5)_3$(0.05 mol%) (doped). The channel width (*W*) and length (*L*) were 1 mm and 0.08 mm, respectively.

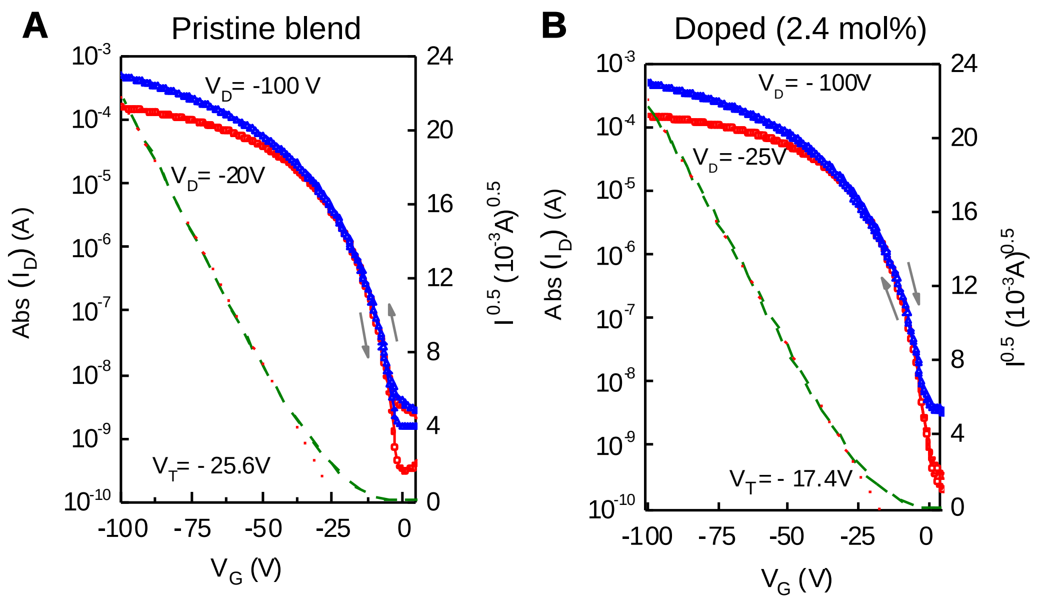

Figure S8. Transfer characteristics measured for OTFTs based on a pristine and a $B(C_6F_5)_3$(2.4 mol%)-doped TIPS-pentacene:PTAA blend layer. The transistor channel width and length were 1 mm and 0.05 mm, respectively.

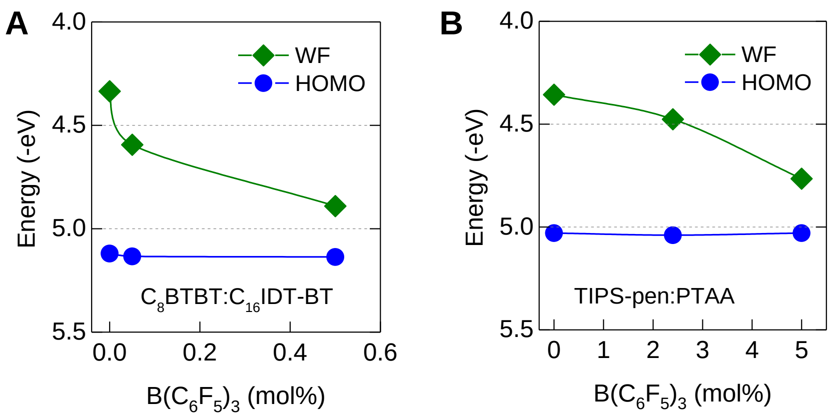

Figure S9. Work function (ϕ) and HOMO energy versus $B(C_6F_5)_3$ loading (mol%) for C_8-BTBT:C_{16}IDT-BT (a), and TIPS-pentacene:PTAA (b).

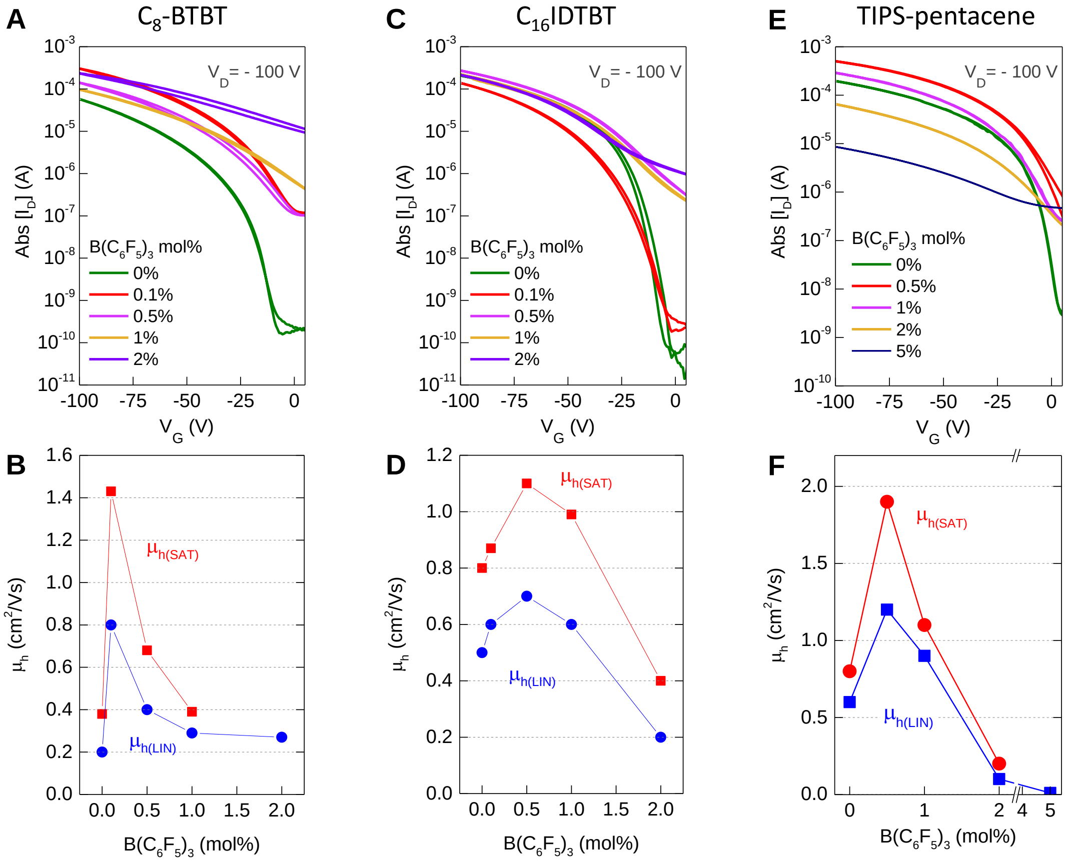

Figure S10. Transfer characteristics (a) and hole mobility calculated from the linear ($\mu_{h(LIN)}$) and saturation ($\mu_{h(SAT)}$) regimes (b) for OTFTs based on pristine and $B(C_6F_5)_3$–doped C_8-BTBT. Transfer characteristics (c) and hole mobility calculated from the linear ($\mu_{h(LIN)}$) and saturation ($\mu_{h(SAT)}$) regimes (d) for OTFTs based on pristine and $B(C_6F_5)_3$–doped C_{16}IDT-BT. Transfer characteristics (e) and hole mobility calculated from the linear ($\mu_{h(LIN)}$) and saturation ($\mu_{h(SAT)}$) regimes (f) for OTFTs based on pristine and $B(C_6F_5)_3$–doped TIPS-pentacene.

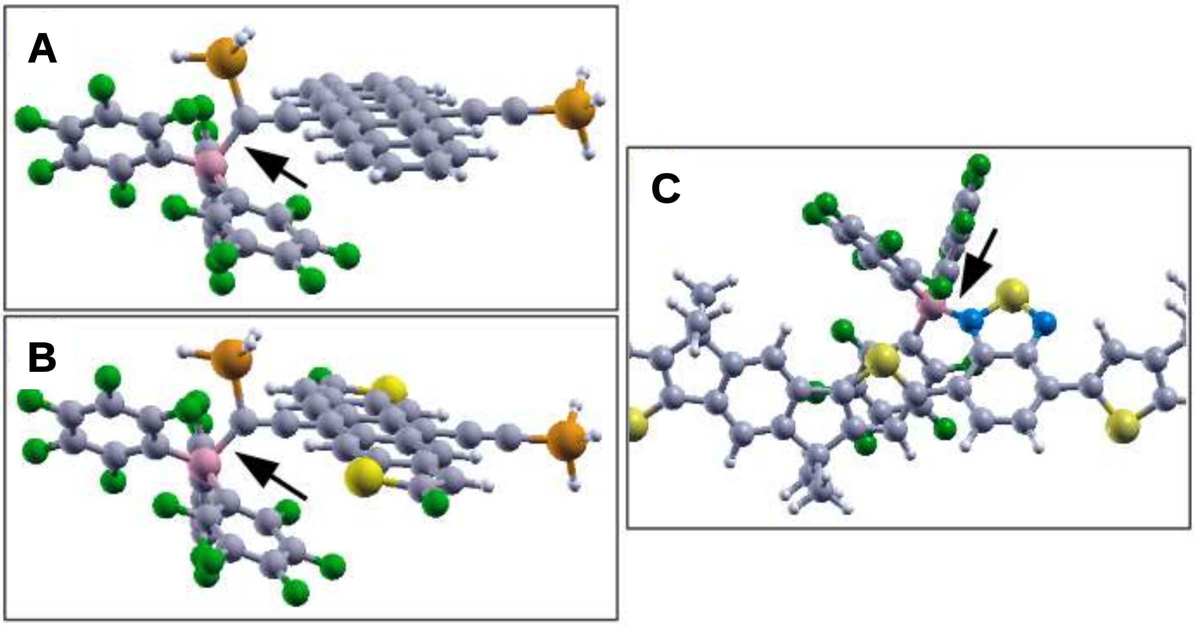

Figure S11. Adduct complexes between a $B(C_6F_5)_3$ molecule and (a) a TIPS-pentacene molecule, (b) a diF-TESADT and (c) a C_{16}IDT-BT polymer chain (C: grey, H: white, B: pink, F: green, Si: orange, S: yellow, N: blue spheres). Their energies are, respectively, 0.02 eV, 0.07 eV and 0.06 eV higher than that of corresponding physisorbed configurations. Arrows in (a) and (b) show the B-C bonds while in (c) the B-N bond. Long side chains are approximated with shorter groups, which account properly for steric effects in the cases considered.
